# Investigating the Mechanism of Sodium Binding to SERT Using Direct Simulations

**DOI:** 10.3389/fncel.2021.673782

**Published:** 2021-05-10

**Authors:** Dániel Szöllősi, Thomas Stockner

**Affiliations:** Institute of Pharmacology, Center for Physiology and Pharmacology, Medical University of Vienna, Vienna, Austria

**Keywords:** human serotonin transporter, sodium binding, kinetics, sodium binding pathway, molecular dynamics simulations, SERT

## Abstract

The serotonin transporter (SERT) terminates neurotransmission by transporting serotonin from the synapse into the pre-synaptic nerve terminal. Altered SERT function leads to several neurological diseases including depression, anxiety, mood disorders, and attention deficit hyperactivity disorders (ADHD). Accordingly SERT is the target for their pharmacological treatments, but also targeted by multiple drugs of abuse. Transport of serotonin by SERT is energized by the transmembrane electrochemical gradient of sodium. We used extensive molecular dynamics simulations to investigate the process of sodium binding to SERT, which is the first step in the transport cycle that leads to serotonin uptake. Comparing data from 51 independent simulations, we find a remarkably well-defined path for sodium entry and could identify two transient binding sites, while observing binding kinetics that are comparable to experimental data. Importantly, the structure and dynamics of the sodium binding sites indicate that sodium binding is accompanied by an induced-fit mechanism that leads to new conformations and reduces local dynamics.

## Introduction

The function of the serotonin transporter (SERT) is to terminate neurotransmission by reuptake of serotonin (5HT) from the synapse into the pre-synaptic nerve terminal. Dysfunction of SERT has been implicated in several neurological diseases including depression, anxiety, mood disorders, and attention deficit hyperactivity disorders (ADHD; Freissmuth et al., 2017). Moreover, drugs of abuse like cocaine or amphetamine interfere with normal SERT function and lead to depletion of the 5HT pools in the pre-synaptic nerve terminal (Hilber et al., 2005).

The structure of SERT has been resolved in the outward-open, outward-occluded, and inward-open conformation (Coleman et al., 2016, 2019). The sodium binding sites were first identified in the homologous bacterial small amino acid transporter LeuT (Yamashita et al., 2005), which also revealed the conserved fold of this transporter family. The substrate binding site (labeled S1) is located in the center of the transporter, halfway through the membrane. The transport cycle leading to 5HT uptake is initiated by binding of substrate and co-transported ions to the outward-open conformation. Full assembly of the transport complex consisting of bound ions and 5HT leads first to 5HT occlusion in the substrate binding site S1, followed by a transition to the inward-open conformation from which substrate and ions are released into the cytosol. Return to the outward-open state is facilitated by binding of a potassium ion or a proton (Nelson and Rudnick, 1979; Hasenhuetl et al., 2016).

An energy source is necessary for facilitating uphill transport, to guaranty directionality and for enabling efficient neurotransmitter clearance by reuptake (Grouleff et al., 2015). SERT belongs to the SLC6 protein family which uses the transmembrane electrochemical gradient of sodium as a primary energy source (Chen et al., 2004). Strict coupling is required between binding of substrate and ions and the key conformational changes of the transport cycle, thereby allowing for efficient transport and initiating transport only once the transport complex has assembled (Tavoulari et al., 2016). Several studies showed that sodium binding stabilizes the outward-open conformation, thereby preventing futile cycling events (Claxton et al., 2010; Zhao and Noskov, 2011; Zhao et al., 2011; Stolzenberg et al., 2015; Tavoulari et al., 2016; Coleman et al., 2019; Li et al., 2019). The high external sodium concentration of 150 mM ensures that the two sodium binding sites are filled, as the K_M_ of sodium for serotonin transport is 25 mM (Quick, 2003). Moreover, a positive cooperativity between sodium and substrate binding was observed that strongly increases the affinity of 5HT to SERT (Chen and Reith, 2003; Hasenhuetl et al., 2018).

**Figure 1:**
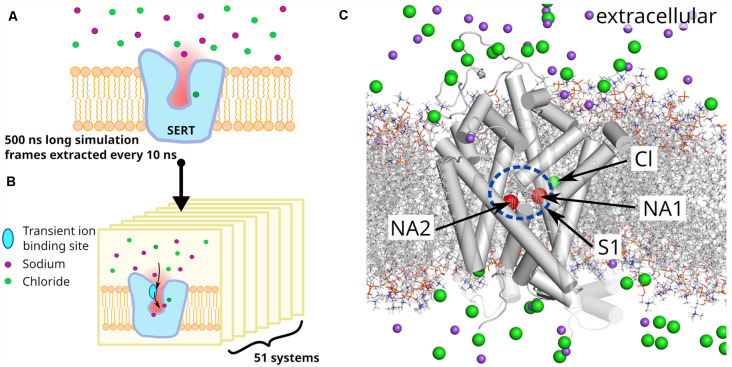
System and system setup. **(A)** A 500 ns long unbiased simulation of serotonin transporter (SERT) in the absence of bound sodium ions was carried out to prepare a set of starting systems. **(B)** Fifty-one snapshots separated by 10 ns were extracted and sodium ions removed, if present in the outer vestibule. All systems were independently simulated for 150 ns. **(C)** Representative starting structure of a selected SERT snapshot, showing sodium (purple) and chloride (green) ions as spheres and highlighting the sodium binding sites NA1 and NA2 by red spheres. The substrate binding site S1 is indicated by a blue ellipse. The lipid membrane is represented by gray dashed lines. NA1 is formed by TM1, TM6 and TM7, NA2 by TM1 and TM8. Temporary binding sites are formed by D326, E493, and E494 from which E493 is part of the extracellular gate (EC) with R104.

The sodium binding sites (referred to as NA1 for site 1 and NA2 for site 2, see Figure 1) of SERT are sodium selective (Felts et al., 2014), a property shared with LeuT (Yamashita et al., 2005; Noskov and Roux, 2008; Zhao and Noskov, 2011). Accordingly, transport is not sustained by other monovalent ions, while mutations of the ion coordinating residues reduce transport activity (Tavoulari et al., 2009; Andersen et al., 2010; Felts et al., 2014). Structures of SLC6 transporters suggest that NA1 and NA2 have distinct roles, with NA1 mainly responsible for substrate binding, while the ion in NA2 is important for stabilization of the outward-open state (Grouleff et al., 2015; Coleman et al., 2019). Solvation of sodium in NA2 is suggested to be important for the transition to the inward-open state (Forrest et al., 2008; Zhao and Noskov, 2011; Borre et al., 2014; Razavi et al., 2017). Simulations investigating the effect of bound ions started with bound ions or investigated the ion-free state (Zhao and Noskov, 2011; Grouleff et al., 2015; Zomot et al., 2015; Razavi et al., 2017; Li et al., 2019).

Kinetic models of the transport cycle, largely build on electrophysiological measurements, predict a sodium association rate constant of 10^6^ to 10^7^ M^−1^s^−1^ (Hasenhuetl et al., 2016, 2018; Burtscher et al., 2019) to the chloride bound outward-open SERT, therefore indicating that at physiological conditions the sodium binding is fast enough (nanosecond to microsecond time-range) to be amendable for direct investigation using molecular dynamics simulation. Starting from a set of 51 SERT structure in the outward-open conformations, extracted from a 0.5 μs long simulation of a sodium free SERT, we simulated sodium binding in 51 independent 150 ns long trajectories (Figure 1), resulting in 8 μs of total simulation time. Sodium binds to at least one of the sodium binding sites in the majority of simulations. We find a transient binding site at the extracellular gate (R104–E493) that acts as initial engagement site with the vestibule of SERT. Sodium enters fast into the vestibule, attracted by a strong negative electrostatic field created by SERT that protrudes into the extracellular medium to facilitate sodium attraction. Before reaching the NA1 and NA2 sites, sodium binds to a second temporary interaction site in the S1 just outside NA1 and NA2. Analyses of the geometries of NA1 and NA2 reveal that their local structures are dynamic in the absence of sodium. The change in geometry of NA1 and NA2 upon sodium binding is reminiscent of an induced-fit mechanism that leads to a more compact, well-defined, and rigid conformation.

## Materials and Methods

We selected the outward-open human SERT crystal structure (PDB ID: 5I71; Coleman et al., 2016) to investigate sodium binding. Missing side chains and the originally absent Cl^−^ ion were positioned using MODELLER 9.20 (Shen and Sali, 2006; Webb and Sali, 2014) creating 100 structures. The best model based on the DOPE score was used for simulations.

The all atom model was converted into a coarse grain representation of the MARTINI force field (Monticelli et al., 2008; de Jong et al., 2013; Wassenaar et al., 2015) and inserted in a 1-palmitoyl-2-oleoylphosphatidylcholine: cholesterol containing membrane (POPC:CHOL 70:30 mol%; van Meer, 1998). The system was solvated in water and 150 mM NaCl. The coarse-grain system was simulated for 1 μs while restraining the protein structure to allow the membrane to accommodate the transporter and to equilibrate around SERT. After membrane equilibration, the coarse-grained system was converted to an all-atom representation (Wassenaar et al., 2014) and the original SERT model replaced the converted protein model to avoid spurious local structural problems induced by the double coordinate conversion of the protocol. Possible atom overlaps between reinserted transporter and the relaxed environment were relaxed using the *membed* procedure (Wolf et al., 2010). We used the amber ff99SB-ILDN force field (Lindorff-Larsen et al., 2010) to describe SERT, ions and the solvent, and Slipid (Jämbeck and Lyubartsev, 2012, 2013) for POPC and cholesterol. Residue Glu508 was protonated as suggested by structural analysis of the SERT crystal structure (Coleman et al., 2016). All simulations were carried out with GROMACS version 2019.2 (Abraham et al., 2015). The final assembled system was energy-minimized and equilibrated in four steps of 2.5 ns, each by stepwise releasing the position restraints (1,000, 100, 10, 1 kJ/mol/nm) that are active on the Cα atoms and the bound Cl^−^. The production run was carried for 500 ns after removing all position restraints. The temperature was maintained at 310 K using the v-rescale (τ = 0.5 ps) thermostat (Bussi et al., 2007), while separately coupling protein, membrane, and solvent. Pressure was maintained at 1 bar using the Parrinello-Rahman barostat (Parrinello and Rahman, 1981) in a semiisotropic manner and applying a coupling constant of 20.1 ps. Long range electrostatic interactions were described using the smooth particle mesh Ewald method (Darden et al., 1993) applying a cutoff of 0.9 nm. The van der Waals interactions were described using the Lennard Jones potentials applying a cutoff of 0.9 nm. Long-range corrections for energy and pressure were applied. Coordinates of all atoms were recorded every 5 ps. The complete set of parameters of the production run can be found in the Supplementary Materials. The resulting trajectory was used as parent trajectory to extract starting structures for the sodium binding simulations, namely taking a snapshot every 10 ns including time = 0 ns, providing 51 starting structures, after removing the sodium ion that entered the substrate binding pocket S1. The resulting 51 systems were simulated for 150 ns with the same simulation parameters as the parent trajectories.

Figures and statistical analyses were generated by the GROMACS package, R, and python scripts using the MD Analysis package, v0.19.2 (Michaud-Agrawal et al., 2011; Gowers et al., 2016). For visualization VMD (Humphrey et al., 1996) v1.9.3 and Pymol v1.8.4 were used.

## Results

### Sodium Binding Kinetics

The aim of this study is to investigate the binding of sodium ions to SERT. To prepare starting structures, we performed a 500 ns long equilibrium simulation of SERT that has a chloride ion bound to the chloride binding site, while the sodium binding sites NA1 and NA2 are empty. SERT is embedded in an equilibrated 1-palmitoyl-2-oleoylphosphatidylcholine: cholesterol (POPC:CHOL) membrane in a 70:30 molar ratio, while the solvent contains 150 mM NaCl. The goal of this initial simulation is to obtain representative SERT conformations. From this 500 ns long equilibrium trajectory, we extract a snapshot every 10 ns, obtaining 51 systems or replicates (Figure 1). Any sodium ion that entered the vestibule during the 500 ns long preparatory simulation is removed and reinserted randomly into the bulk solvent. An independent simulation of 150 ns was carried out for each of these 51 systems.

**Figure 2:**
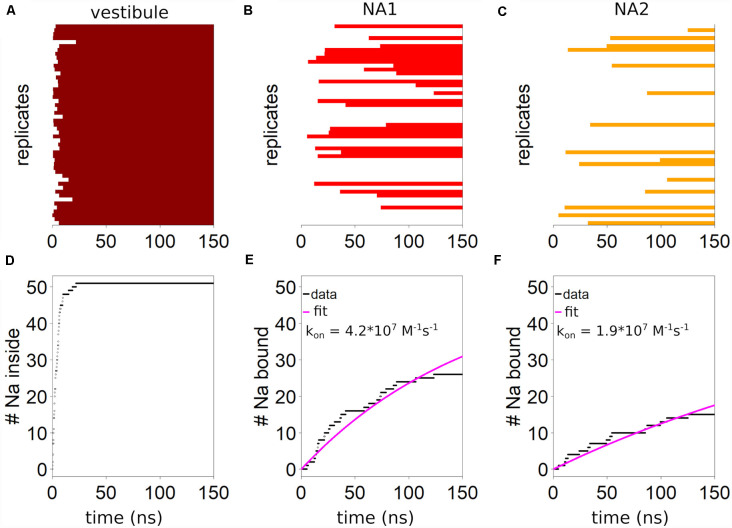
Kinetics of sodium binding. The presence of at least one sodium ion **(A)** in the vestibule is indicated by a horizontal dark red bar for the 51 simulations or replicates. The outer salt bridge between R104 and E493 is defined as the boundary for being within the outer vestibule. **(B)** Binding of sodium to NA1 is indicated by red horizontal bars. **(C)** The presence of sodium in NA2 is symbolized by orange bars. Cumulative number of trajectories (replicates) showing **(D)** sodium ions entering the vestibule, **(E)** bind to NA1 or **(F)** to NA2. Estimation of the first order rate constant of sodium binding by fitting an exponential equation of the form: y = A·(51-e^−bt^), with the pre-factor constrained to 51 assuming all conformations are capable of binding.

We define the extracellular salt bridge between residue R104 on TM1 and residue E493 on TM10 as the structural cut-off for identifying any sodium ion to reside within the vestibule, as once passing this salt bridge, sodium ions typically remain within the vestibule. The salt bridge is part of the extracellular gate (EC) in the SLC6 family (Yamashita et al., 2005), which in SERT consists of the salt bridge (R104-E493) and the hydrophobic lid (F335, I172, Y176) that together seal the S1 from the extracellular side if SERT is inward-facing (Coleman et al., 2016). These simulations show rapid sodium entry into the outer vestibule of SERT with a mono-exponential time dependency and a half-value entry time of 5.7 ns (Figure 2A), and every system contains at least one sodium ion in the outer vestibule after 22.2 ns (Figure 2D).

Binding to NA1 and NA2 is slower. Sodium enters NA1 (Figure 2B) in 27 of the 51 simulations (150 ns each), while binding to NA2 is in 15 simulations (Figure 2C). Figure 2 shows the binding kinetics of the complete dataset. By fitting a mono-exponential curve to the data we find a binding constant of 4.2·10^7^ M^−1^s^−1^ for NA1 (Figure 2E) and 1.9·10^7^ M^−1^s^−1^ for NA2 (Figure 2F). The first order rate constant for sodium binding to both NA1 and NA2 is 9.8·10^6^ M^−1^s^−1^. The rate constants detected in these simulations are in reasonable agreement with the experimental data-derived kinetic models that predict a sodium binding rate constant to both sodium binding sites between 10^6^ and 10^7^ M^−1^s^−1^ (Hasenhuetl et al., 2016, 2018; Burtscher et al., 2019). Interestingly, in eight out of the nine simulations when sodium binds to both sodium binding sites, NA2 is filled before NA1. In the majority of trajectories which display sodium binding to NA2, this event is followed by sodium binding to NA1. In contrast, initial binding of sodium to NA1 is followed rarely (1 case) by sodium binding to NA2, suggesting that binding of sodium to one sodium binding site might affect binding to the other sodium binding site.

The systems which bind sodium ions are randomly distributed among the 51 trajectories, therefore indicating that the conformations extracted from the 500 ns long parent simulation do not have a conformational bias. A structural change occurring during the preparatory 500 ns simulation that could affect and/or modulate sodium binding would also create a distinct pattern of sodium binding events among the 51 trajectories that deviates from a random distribution. We observe chloride unbinding in a few simulations, whereby it is leaving its binding site through the empty NA1 site, followed by fast diffusion through the vestibule into the extracellular bulk solvent. The binding of sodium to NA1 could not be observed in any of these systems, suggesting that the presence of a bound chloride is necessary for sodium binding to NA1.

### The Path of Sodium Through the Outer Vestibule

**Figure 3:**
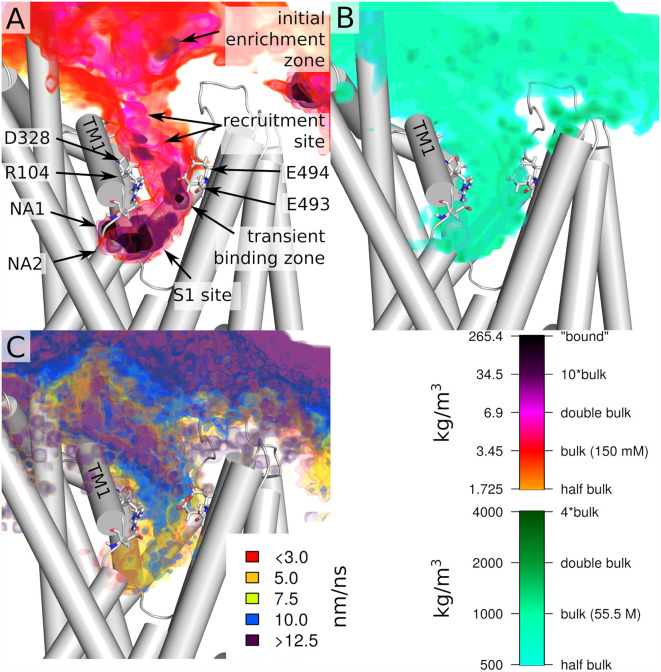
Overview of the entry path of sodium ions. **(A)** Spatial density of sodium ion and **(B)** water at the protein extracellular interface and in the outer vestibule. **(C)** Velocity of sodium ion movements (displacement) at the extracellular interface and in the outer vestibule. All values are shown as colored volumes together with the starting structure of the first replica. Values are averages of all 51 trajectories, which were initially fitted by their Cα atoms. Trajectories were analyzed every 5 ps for the spatial densities and every 10 ps for displacement. The spatial grids have a resolution of 0.1 nm.

Next, we investigate the path that sodium takes through the outer vestibule. For the analysis, we fit all trajectories to a reference frame (starting conformation of the first trajectory) using Cα atoms, which allows us to average the entire dataset. To identify the areas of frequent sodium encounters, we create a 3D spatial density map of sodium positions averaged over the 51 trajectories, which therefore is a histogram of encountered sodium positions over the entire dataset (Figure 3A). In the extracellular space, this histogram represents an average density of the randomly diffusing sodium ions, in the outer vestibule the density is associated with the path that sodium takes to reach the S1, while in the S1 the density is linked to the areas of most frequent encounters of sodium before binding to NA1 or NA2. We find that the sodium ions are attracted to SERT at its extracellular site, as the sodium concentration above the outer vestibule is larger than the average density in the extracellular solution (initial enrichment zone). An initial recruitment zone exists in the extracellular vestibule above the outer salt bridge. It is located next to residue D328 that attracts sodium ions by its negative charge, thereby locally increasing the concentration and thus promoting sodium binding to SERT. Experimental data have shown that mutations of D328 affect sodium binding (Kortagere et al., 2013). Directly at the salt bridge between R104 and E493 (Figure 3A) at the EC gate a transient sodium binding site exist, to which the ions typically remain bound for a short period of time before moving further towards the S1 substrate binding site (Figure 3). The width of the largest sodium density (Figure 3A) is comparable to the size of a sodium ion, while the density indicates that the transient binding site is extended vertically along the outer vestibule, suggesting that this dynamic binding zone attracts sodium ions for promoting their transition towards the substrate binding site S1. This sodium binding zone is dynamic, as: (i) sodium ions are not bound to a specific position similar to NA1 and NA2, but move within this zone and (ii) because sodium ions remain bound for only a short period of time, consistent with a low affinity site.

The largest density of sodium ions is observed in the S1 and in the NA1 and NA2 sites. We find that sodium ions reside for some time just outside the NA1 and NA2 binding sites before final binding. Important to note that in two simulations a third sodium was present at the S1 site. This increasing sodium density towards NA1 and NA2 is indicative of a force generated by SERT that attracts sodium ions. Figure 3B shows water density and therefore indicates the size of the outer vestibule. A comparison between the density of sodium and the area occupied by water shows that sodium spreads through most of the volume of the outer vestibule. An important exception seems to be the region surrounding R104. While the positive charge of the arginine will create a repulsive force acting on the positively charged sodium ions, the extracellular salt-bridge partner E493 and the adjacent E494 are highly attractive and form the temporary sodium binding site. While reaching to most of the vestibule volume, the density of sodium occupancy suggests that sodium follows a preferred path through the vestibule.

Complementary to the relative sodium density, we quantified the average displacement of sodium ions by taking a snapshot every 10 ps, averaged over the 51 trajectories. Figure 3C shows the average displacement of sodium ions mapped to the starting position of the first of the two consecutive frames. In the vestibule, the average displacement is smaller as compared to the extracellular solutions, showing that the velocity of sodium diffusion is lower within the vestibule. Sodium displacement is not equally reduced throughout the outer vestibule. The regions of very low displacement of 7.5 nm/ns or smaller are associated with the regions of high sodium density because a sodium ion remains in this region of higher density for a longer time. A comparison between sodium density (Figure 3A) and average sodium displacement (Figure 3C) indicates that high sodium density and small displacements are not fully correlated. This is most evident at the transient sodium binding site at the EC gate and in the region connecting it to the S1. The upper part of the high sodium density at the outer gate shows high sodium displacement, which is consistent with repeated fast sodium entering and leaving the region. In contrast, at the lower end of the transient sodium binding site at the EC gate, the average sodium displacement is very low (5–7.5 nm/ns), indicative of a more specific ion binding site, which nevertheless remains transient. This data indicates that sodium becomes first dynamically recruited from the extracellular medium and is guided towards the transient binding site at the EC gate. The region between this transient sodium binding site and the S1 substrate binding site shows an increased displacement of 10 nm/ns. The same region overlaps partially with high sodium density in the S1, showing that at the upper end of the S1 the average movement of sodium is relatively fast. In contrast to these fast movements, in the S1 regions juxtaposed to NA1 and NA2, movements of sodium ions are below 3 nm/ns, indicative of an increasingly strong attracted sodium. Sodium ions remain associated to these temporary sites for some time before entering NA1 or NA2. Similar sites have been identified in the bacterial homolog LeuT (Zomot et al., 2015).

### Binding of Sodium to the Binding Sites NA1 and NA2

**Figure 4:**
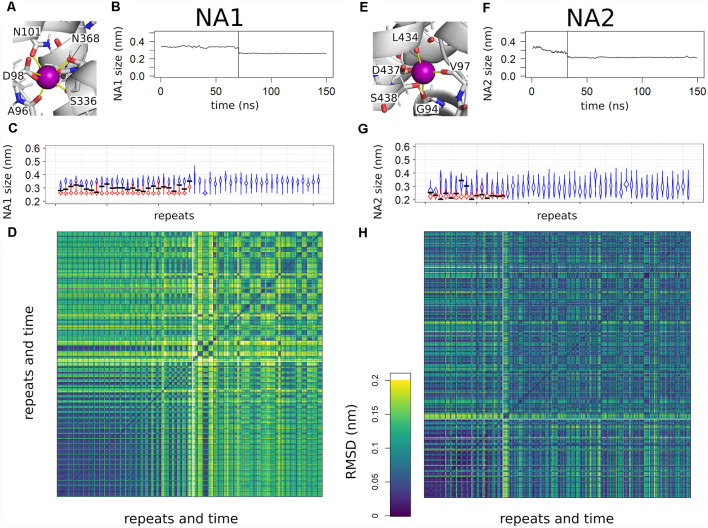
Overview of sodium binding to NA1 and NA2. **(A,E)** Close up to NA1 and NA2. Distances used for the compactness measurement are highlighted by yellow lines. The same binding atoms are also used for the root mean square deviation (RMSD) analyses. **(B,F)** A representative example for the change in the compactness upon sodium binding. The time point of sodium entering NA1 or NA2 is highlighted by a vertical line. Curves are smoothed by a 1 ns running average. **(C,G)** Violin plots of the binding site compactness for all simulations sorted according to the time point of sodium binding to NA1 or NA2, respectively. In case of a sodium binding event, the trajectory was divided at the time of sodium binding. The violin in blue represents the pre-bound state and the violin in red shows the compactness after binding. The compactness at the time point of sodium binding is highlighted by a horizontal black line. The temporal resolution of the distance analysis was 100 ps. **(D,H)** RMSD analysis of all simulations sorted as in panels **(C,G)** of the atoms used for measuring NA1 and NA2 compactness. The boundaries between individual simulations are indicated by a black grid, while the white lines are used to separate simulations that show sodium binding from simulations in which sodium does not bind to SERT. Temporal resolution for the RMSD matrix was 10 ns.

Sodium ions entering NA1 and NA2 are the final steps of binding to SERT. Figure 2 shows that the binding kinetics are similar, the association rate to NA1 being faster than to NA2. The binding of ligands to proteins is typically associated with structural changes. The mode of these structural changes can range from conformational selection (Monod et al., 1965), where a pre-existing conformation is selected by the ligand, to an induced-fit mechanism (Koshland et al., 1966), in which the protein assumes a new conformation upon ligand binding. To identify and quantify the structural change in NA1 and NA2 upon sodium binding, we defined a structural measure of the sodium binding site and correlated these with sodium association. Sodium is coordinated by several oxygen atoms from the SERT backbone and side chains. Figures 4A,E show a structural legend of the used measures, which consists of the average distance between all sodium coordinating atoms of SERT (NA1: A96-O, D98-CG, N101-OD, S336-O, S336-OH, N368-OD; NA2: G94-O, V97-O, and L434-O) and their center of mass. This average distance represents a quantification of the compactness of NA1 and NA2. The center of mass coincides well with the position of bound sodium ions if present.

Figures 4B,F show a representative time course of the compactness of NA1 and NA2 of one simulation. Respective plots for all other systems that bind sodium ions can be found in Supplementary Figures 1,2. The average distance between oxygen atoms is larger before sodium binding, while sodium binding marks a steep drop in the average distance. Figures 4C,G show an overview of all simulations as a violin plot, which summarizes all observed distances and represents the relative probability by the respective width. Trajectories are separated between pre- (blue) and post- (red) sodium binding, if sodium enters the sodium binding site, with the black bar indicating the compactness at the moment of sodium binding. The trajectories are sorted according to the time point of sodium entering the sodium binding site according to Figure 2. The NA1 is on average by 0.1 nm more open in the absence of sodium (Figure 4C), while the variability of the compactness of the NA1 site is larger in the absence of sodium as compared to the sodium-bound state. The compactness of NA1 at the moment of sodium entry (black bar) is between the average pre- and post-sodium binding state. The range of compactness values at the moment of sodium entry indicates that no specific geometry or compactness of the sodium binding site(s) is required. Importantly, the values indicate that sodium enters when the apo state is particularly compact. Similar observations also apply to NA2 (Figure 4G), as the binding site is more compact in the presence of sodium, while the time point of sodium entry is associated with an NA2 size that is small for the apo state.

To more directly compare the geometries of the sodium binding sites across the 51 trajectories, we measured the root mean square deviation (RMSD) of the same sodium coordinating residues in Cartesian space. Figures 4D,H show a comparison of all simulations vs. all simulations, sorted according to Figures 4C,G. The squares on the diagonal of the matrix represent a structural comparison of structures of one simulation to all other structures of the same simulation, while the off-diagonal squares measure similarity between different simulations. The big dark violet block at the lower end of Figures 4D,H shows very similar values along the diagonal and for the off-diagonal squares after sodium binding. This big block includes all trajectories that show sodium binding. Sodium enters the binding site right at the beginning of the simulations in the first trajectories at the lower-left corner, while the moment of sodium entry continues shifting to later time points according to trajectory sorting. The respective time points are shown in Figure 2. RMSD values consistently change from high values to low values, once sodium binds. The RMSD plot complements the compactness measure and shows that the same local sodium bound conformation keep being reached after binding.

The situation is different for the simulations, which did not show sodium binding: the RMSD values show that the geometry is variable (off-diagonal elements have low or high RMSD values) with some trajectories being similar to each other while differing from others. Almost all trajectories differ from the sodium-bound conformation, the few exceptions showing that the sodium-bound-like geometry can be reached in the absence of sodium, but also indicates that such a state is rare. A very similar pattern can be observed for NA2, as binding of sodium promotes a well-defined conformation, while sodium-free state samples multiple conformations. However, the overall variability is smaller.

### Electric Field in the Vestibule

**Figure 5:**
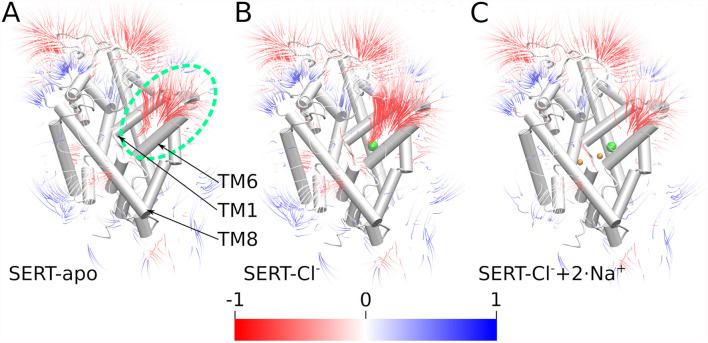
Electrostatic gradients and fields. Electrostatic potential field lines solved by the adaptive Poisson-Boltzmann solver (APBS) of the outward-open SERT of **(A)** the apo state, **(B)** SERT with bound Cl^−^, **(C)** SERT with bound Cl^−^ and Na^+^ ions; the green dashed ellipse highlights the outer vestibule. Field lines are colored by potential with a color gradient form red through white till blue representing a scale from −1 to 1 (kT/e). Field strength more negative than −1 is red while values above 1 are blue.

The density analysis (Figure 3) shows an above-average sodium density in the outer vestibule and in the adjacent solvent. To investigate if SERT would generate a negative electrostatic field that could attract the positively charged sodium ions, we quantified the electrostatic potential and the field lines generated by SERT using the adaptive Poisson-Boltzmann solver (APBS; Baker et al., 2001; Dolinsky et al., 2004). Consistent with the increased local sodium density, SERT creates a strong negative electrostatic potential in the outer vestibule with the field lines reaching into the solvent adjacent to the entry site at the outer vestibule (Figure 5). The strength of the field is a function of the ion-binding state of SERT. The electrostatic field lines and thus the field gradient reaches from the S1 to the extracellular solvent in the apo state of SERT (Figure 5A). The field is even stronger in the presence of the chloride ion (Figure 5B), while in the presence of sodium ions bound to NA1 and NA2 the gradient of the electrostatic field is weaker. These data indicate that SERT exerts an attractive force for sodium ions to reach the S1 and for binding to NA1 and NA2. The residual negative potential and the residual field gradient after sodium binding indicates that SERT can still attract 5HT by electrostatic interactions.

## Discussion

The binding and co-transport of extracellular sodium into the cell and the movement downhill its electrochemical gradient is the primary determinant for driving the transport cycle of SERT (Chen et al., 2004). This electrochemical gradient of sodium leads under physiological conditions to the transport of 5HT into cells even during cellular conditions where the transport is uphill the 5HT chemical gradient (Grouleff et al., 2015). The binding of sodium ions stabilizes the outward-open conformation of SERT (Claxton et al., 2010; Zhao and Noskov, 2011; Zhao et al., 2011; Stolzenberg et al., 2015; Tavoulari et al., 2016; Coleman et al., 2019; Li et al., 2019) and strongly increases the affinity of the substrate 5HT, while transport occurs only once the complex between SERT, 5HT and the co-transported ions is established (Masson et al., 1999; Felts et al., 2014; Tavoulari et al., 2016). Stabilization of substrate in the S1 was linked to NA1, as in LeuT the substrate interacts directly with the sodium ion in NA1 (Yamashita et al., 2005; Grouleff et al., 2015; Coleman et al., 2019), while in the monoamine transporters, the sodium ion in NA1 positions the sidechain of the adjacent aspartate (D98 in SERT) to interact with the amino groups of the substrate (Wang et al., 2015; Coleman et al., 2016, 2019). The main role of the sodium ion bound to NA2 is to stabilize the closed intracellular gate and thus the outward-open conformation (Zhao and Noskov, 2011; Zhao et al., 2012; Khelashvili et al., 2015; Tavoulari et al., 2016; Razavi et al., 2017; LeVine et al., 2018).

In this study, we investigate the first step of the transport cycle, which is the binding of sodium ions to the outward-open conformation of SERT in the presence of chloride ions, as experimental data indicate that chloride remains continuously bound throughout the transport cycle (Buchmayer et al., 2013; Hasenhuetl et al., 2016). We find that the structure of SERT is optimized for an efficient recruitment of sodium ions into the open vestibule and observe an association rate constant (k_on_) of 9.8·10^6^ M^−1^s^−1^, which is in good agreement with experimentally observed rate constants (Hasenhuetl et al., 2016, 2018; Burtscher et al., 2019). The fast binding is induced by the strong electrostatic field of SERT, which has a negative sign in the outer vestibule and guides the positively charged sodium ions into the S1. The computationally identified rate constant should be considered an upper estimate, because in simulations the starting structure of SERT is already in a sodium binding competent conformation, while the experimental procedure of the electrophysiological measurements provides a more complex readout, which is convoluted by conformational changes of SERT. The situation is less clear for LeuT, as sodium, which has a low mM affinity for LeuT (Zhao et al., 2010), was stably bound to NA2 in most simulations (Zhao et al., 2012; Tavoulari et al., 2016), but was also found to dissociate from LeuT through the outer vestibule in one study (Zomot et al., 2015).

The binding of sodium ions to SERT is a multilayered process: sodium ions become initially attracted to a binding zone outside the extracellular salt bridge that serves as an initial recruitment zone for positively charged ions thereby increasing their local concentration. The most important residue in this recruitment zone is D328. A mutation of this residue was shown to decrease the affinity of amphetamines and to reduce amphetamine-mediated efflux (Kortagere et al., 2013). Mutation of the corresponding residue in the dopamine transporter (D313) was shown to decrease the affinity for dopamine, which was suggested to be an indirect effect that is caused by a reduced accessibility of sodium ions for reaching the sodium binding sites (Chen and Reith, 2003).

At the extracellular gate, we find a transient sodium binding site, which shows a high propensity to attract sodium ions that were initially recruited to the zone next to D328. Despite simulations showing high local ion density, sodium ions did not remain bound for very long to this site next to the glutamate residues 493 and 494. A weak sodium binding affinity seems mechanistic important because preventing any interference with proper assembly of the transport complex consisting of ion- and 5HT-bound SERT. If in contrast, this site would represent a strong sodium binding site halfway to the S1, it would be detrimental for transport, because preventing or at least slowing the processes of sodium and 5HT binding. Importantly, the narrow shape of the outer vestibule at the extracellular gate provides sodium ions with only two escape routes: unbinding towards the extracellular space or proceeding towards S1. The direction of the electrostatic field lines pointing towards the more negative S1 ensures that sodium ions move more likely towards NA1 and NA2 if the sites are not yet sodium bound.

In the last step before stably binding to SERT, sodium ions associate with a second transient binding site in the S1 juxtaposed to the NA1 and the NA2 sites. A comparable behavior was observed in LeuT (Zomot et al., 2015). This second transient binding site coincides with the position of positively charged nitrogen atoms of substrates and inhibitors as observed in structures of dDAT and hSERT (Wang et al., 2015; Coleman et al., 2016), suggesting that it has a dual role: (i) to serve as a transient site to efficiently recruit sodium ions; and (ii) to form an interaction site for the positive changed nitrogen of the monoamine substrate 5HT.

Structural changes induced by ligand binding have frequently been described as following an induced-fit mechanism (Koshland et al., 1966) or conformation selection (Monod et al., 1965), which represent extreme views of structural adaptations of proteins to the presence of ligands. The conformational changes associated with sodium binding to NA1 and NA2 suggest an overall induced-fit mechanism (Figure 4), as NA1 and NA2 are more compact in the presence of sodium. The situation is particularly clear for NA1, which is very dynamic and wide open in the absence of sodium, but also NA2 shows a comparable behavior. The time point of sodium association correlates with a very compact geometry of NA1 in the absence of sodium. Such a selective binding process would be consistent with a conformational selection as sodium ions seem to select a particular conformation for their association. At the same time, the NA1 compacts further once a sodium ion is bound. This becomes apparent in Figure 4 as NA1 becomes more compact after sodium binding, thus a clear indication of an induced-fit effect. Together these data indicate that the structural arrangement of NA1 might be a combination of an initial conformational selection followed by an induced-fit condensation. The driving forces for compacting the NA1 site are most likely electrostatic attractions between sodium and the coordinating oxygen atoms of SERT that carry partial negative charges. Water molecules occupy NA1 and NA2 if their site are not binding a sodium ion. The neutral water molecule, which is slightly bigger than a sodium ion, cannot lead to the same electrostatic attractions, while also unable to form hydrogen bonds with all potential interaction partners. For the initial conformational selection, similar arguments may apply: (i) initial stabilization of sodium is more efficient, if the site is more compact, (ii) while the presence of water molecules might be energetically less favorable if the site is more compact.

Beyond the core three carbonyl backbone oxygen atoms of residues G94, V97, and L434 of NA2, the side chains of D437 and S438 contribute to complete the NA2 site. Their interaction with the bound sodium ion is more dynamic and can be replaced by a water-bridged interaction. The conformation of S438 is associated with sodium entry, because the side chain needs to rotate for allowing initial sodium association, thereby forming a small barrier which could explain the slower association rate as compared to NA1. The side chain of residue D437 is partially solvated in our simulations as also observed in other studies (Zhao and Noskov, 2011; Khelashvili et al., 2015; Tavoulari et al., 2016; Razavi et al., 2017; LeVine et al., 2018). These water molecules reach residue D437 from the intracellular side; it is, therefore, conceivable that water interactions with and the dynamics of the side chain of residue D437 are associated with the transition towards the inward-facing state. Both residues (D437 and S438) are not essential for protein function as their mutation does not affect the surface expression of SERT, while transport activity is reduced between 15% and 93%, depending on the identity of the mutation (Felts et al., 2014). These data therefore suggest that the three backbone carbonyl oxygen atoms are essential for sodium binding, while the side chains of D437 and S438 have a secondary role in NA2 function.

In conclusion, our large-scale simulation approach to directly study the association of sodium ions reveal that SERT creates a strong electrostatic field that reaches into the extracellular milieu and attracts positively charged ions towards their binding sites. The data show that specific sites for sodium interactions exist within the outer vestibule which are transient enough to support efficient recruitment and transition of sodium ions. The binding of the sodium ions to the NA1 and the NA2 sites is associated with local structural changes that consist of an initial conformation selection followed by an electrostatic driven induced-fit process.

## Data Availability Statement

The raw data supporting the conclusions of this article will be made available by the authors, without undue reservation.

## Author Contributions

DS and TS contributed in the conception of the work, the design of data analysis, the interpretation of the results, and drafted the manuscript. DS performed the simulations, data collection, and developed the analysis code. TS provided funding and supervised the work. All authors contributed to the article and approved the submitted version.

## Conflict of Interest

The authors declare that the research was conducted in the absence of any commercial or financial relationships that could be construed as a potential conflict of interest.
